# Protocol for isolating and characterizing effector functions of murine bone marrow-derived eosinophils following bacterial challenge

**DOI:** 10.1016/j.xpro.2025.104104

**Published:** 2025-10-10

**Authors:** Katelyn M. Parrish, Tyler L. Williams, Monica C. Gestal

**Affiliations:** 1 Department Microbiology and Immunology, Louisiana State University Health Sciences Center at Shreveport, Shreveport, LA 71103, USA

**Keywords:** Cell Biology, Cell isolation, Cell-based Assays, Immunology, Microbiology, Molecular Biology

## Abstract

Understanding eosinophil-associated mechanisms, especially in the context of infection, has been steadily increasing. Here, we present a protocol for isolating and differentiating murine bone marrow-derived eosinophils (bmEos) followed by multi-well plate assays to evaluate eosinophilic responses to bacterial challenge. We describe steps for evaluating eosinophil effector functions including cytotoxicity, bactericidal activity, and cytokine production through multiplex ELISA panels. We use a *Bordetella bronchiseptica* infection model, but this approach can be modified for an array of microorganisms and different experimental settings.

For complete details on the use and execution of this protocol, please refer to First et al.^1^

## Highlights

• Steps for differentiating mouse bone marrow progenitor cells into eosinophils (bmEos)
• Instructions on analyzing purity of differentiated bmEos via flow cytometry
• Guidance on preparing *B. bronchiseptica* inoculums for bmEos infection assays
• Procedures for functional *in vitro* assays following bmEos bacterial challenge

## Abstract (editor highlights)

Publisher’s note: Undertaking any experimental protocol requires adherence to local institutional guidelines for laboratory safety and ethics.

## Graphical abstract

**Figure:**
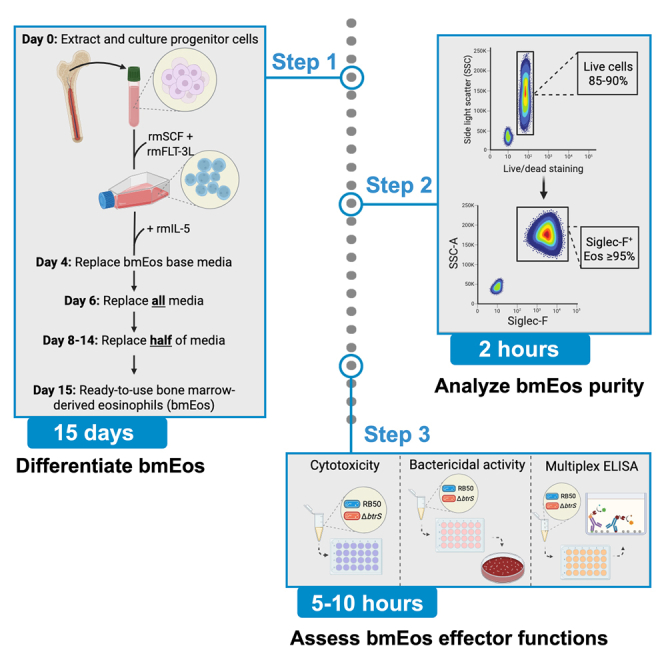

## Before you begin

Eosinophils are involved in viral,^2^^,^^3^^,^^4^ fungal,^5^^,^^6^^,^^7^^,^^8^^,^^9^^,^^10^ and bacterial^1^^,^^11^^,^^12^^,^^13^^,^^14^ immune responses. The antibacterial properties of eosinophils, demonstrated over 50 years ago, extend to direct bacterial killing.^15^^,^^16^^,^^17^ *Staphylococcus aureus* is well-established to be associated with atopic dermatitis and eczema.^18^ Interestingly, multiple *S. aureus* toxins have been shown to selectively activate and stimulate eosinophils,^19^^,^^20^^,^^21^ implicating their involvement in eosinophil-mediated pathologies. It has also been shown that during *Clostridium difficile* infection, recruitment of eosinophils correlates with better prognosis.^22^ During *Helicobacter pylori* infections of the gastrointestinal (GI) tract, eosinophils are important mediators of immunosuppressive strategies employed by *H. pylori*,^23^ emphasizing the intricate involvement of eosinophils in mucosal immune responses which are increasing in recognition as ongoing investigations further explore these connections.

The protocol below describes specific steps for differentiation of eosinophils from primary murine bone marrow-progenitors. This procedure, derived from the published protocol by Mai et al. (2021),^24^ includes additional tips to evaluate effector functions. Here, we standardized experimental settings for investigating the bactericidal activities of eosinophils, using *Bordetella* spp. as a model. This protocol can be adapted to work with other bacterial pathogens. Some tips and guidelines on making these protocol adjustments can be found in “Alternatives” sections.

It is important to mention that mouse eosinophils differ from human eosinophils. However, the vast majority of similarities and the easy access to murine eosinophils^25^ allows for the use of these cells in mechanistic investigations, that can be later be ask utilizing human eosinophils. Additionally, ensure you have the appropriate mice before beginning this protocol. We recommend using BALB/c mice which were used for the experiments included in this protocol as well as our published data.^1^ Differences in the genetic background of wildtype and non-wildtype mouse strains are discussed further in the “expected outcomes” section.

### Innovation

Eosinophils are granulocytes characterized by a short circulatory half-life and extended tissue residence,^26^ particularly in mucosal sites, being the most commonly investigated the Peyer’s patches.^27^^,^^28^ While extensively researched in the context of asthma,^29^^,^^30^^,^^31^ allergies,^32^^,^^33^ eosinophilic esophagitis,^34^ and fungal^35^ or parasitic infections,^36^^,^^37^ the contribution of eosinophils to immune responses against non-fungal or parasitic pathogens has become a subject of recent interest.^1^^,^^38^^,^^39^ The increased research on eosinophils challenges our current understanding of these cells and even traditional knowledge is being disputed.

While the GI tract^27^^,^^28^^,^^40^^,^^41^ and skin^42^^,^^43^^,^^44^ are the main targets for studying the immunomodulatory functions of eosinophils, the primary focus of our studies is to mechanistically investigate the role of eosinophils in the context of respiratory bacterial infections. However, despite the correlative evidence that some bacterial pathogens (e.g., *Bordetella pertussis*) can induce asthma,^45^ less research focuses on the involvement of eosinophils during respiratory infections. Using *Bordetella bronchiseptica*, the evolutionary ancestor of *B. pertussis*,^46^^,^^47^ and a natural murine model of chronic respiratory disease,^48^ we identified a critical role for eosinophils in contributing to mucosal immune responses during infection.^1^^,^^14^ This evolving landscape of eosinophil research spans a diverse array of pathogens and immune responses, not only challenging established paradigms but also uncovering promising facets about their immunological roles. As we deepen into the intricacies of eosinophil biology, we anticipate unlocking exciting insights that will broaden our understanding of immune responses and pave the way for innovative therapeutic strategies.

### Institutional permissions

All mouse procedures during this protocol were performed in accordance with IACUC regulatory standards and institutional guidelines (AUP:21-031, AUP:22-031, APU: 24-036). Mice were originally purchased from Jackson Laboratories (Bar Harbor, ME) for breeding in-house. Our breeding colonies were kept under the care of the employees and veterinarians of the Animal Care Facility within Louisiana State University Health Sciences Center at Shreveport, which is accredited by the Association for Assessment and Accreditation of Laboratory Animal Care (AAALAC). All mice are maintained under a 12-h light/dark (7 am-7 pm) cycle in pathogen-free conditions at all times. For genotype maintenance of each mouse strain, each breeding colony is regenerated after 5 generations. A genotyping database is kept in electronic format as well as in paper format, consisting of all necessary information for each individual mouse, including genotype, age, gender, and an experiment log code, based on the ear tag numbers, indicating the procedures in which they were used as well as the results generated.

Institutional and biosafety guidelines and procedures for handling a BSL-2 pathogen, in our case *Bordetella* spp. (B20-004), should be in place. If this protocol is adapted for other microorganism, the biosafety guidelines should be adjusted as required by the committee, and pilot experiments assessing optimal multiplicity of infection are highly encouraged.

### Preparation of bmEos cytokine stocks

**Timing: 15 min**

1. rmFLT-3L and rmSCF preparation.
  a. Briefly centrifuge each lyophilized cytokine prior to reconstitution.
  b. Reconstitute rmFLT-3L and rmSCF in 1 mL of 0.1% BSA diluted in PBS.
  c. Store reconstituted rmFLT-3L and rmSCF in 500 μL aliquots at −80°C until use.
  d. Sterilize all prepared reagents using a filtration system with a 0.22 μm pore size.
2. rmIL-5 preparation
  a. Briefly centrifuge each lyophilized cytokine prior to reconstitution.
  b. Reconstitute rmIL-5 in 1 mL of 0.1% BSA diluted in PBS.
  c. Store the reconstituted rmIL-5 in 100 μL aliquots at −80°C until use.

### Preparation and seeding of bmEos for multi-well plate assays

**Timing: 60 min**

3. Prepare a 96-well plate by seeding 100 μL of approximately 10^4^–10^5^ bmEos into each well.

***Note:*** Eosinophils remain in suspension and do not attach to cell culture flasks, so they do not require trypsinization prior to harvesting.

**CRITICAL:** Seeding of the multi-well plate should be done at least 24 h prior to performing infection assays to allow the bmEos to return to a basal, resting state.

4. Count the number of eosinophils using an automatic or manual hemocytometer in order to prepare an accurate bacterial MOI for the assay.

***Note:*** We recommend avoiding using concentrations that are above 10^6^ because if eosinophils are too crowded, the activation status can be affected.

### Preparation of agar plates for bacterial culture and CFU

**Timing: 8–12 h**

5. Prepare agar for bacterial plating prior to beginning experimental assays
  a. Dissolve 30 g/L of Bordet-Gengou (BG) agar base using deionized water in a sterilized, heat-resistant glass bottle. Add 10 g/L of 100% glycerol and mix using a magnetic stirring platform.
  b. Sterilize the prepared media using an autoclave cycle at 121°C for 15 min.
  c. Following sterilization, monitor the heated agar closely until the temperature of the media reaches 45°C–50°C.
  d. Aseptically add 1 mL of streptomycin (20 mg/mL) to each bottle of agar, and 10%–15% defibrinated sheep’s blood that has been pre-warmed to 27°C–37°C. Gently swirl to mix until homogenized, avoiding bubbles.
    ***Note:*** Addition of streptomycin to BG agar (BGS) selects for *Bordetella* spp. strains and limits contaminants.
    **CRITICAL:** Do not add the streptomycin or blood prior to sterilizing the agar solution. The sterilization cycle will degrade the antibiotic and destroy the blood components needed for bacterial growth.
  e. Pour approximately 20 mL of prepared agar into 100 mm x 15 mm petri dishes under aseptic conditions, using a Bunsen burner flame to avoid contamination.
  f. Allow the agar plates to cool and harden at room temperature before use. Once the agar plates have cooled completely to room temperature, they can be stored at 4°C for up to one month.

### Preparation of bacterial liquid cultures for inoculums

**Timing: 30 min**

6. Prepare bacterial liquid cultures prior to beginning experimental assays.
  a. For culturing *Bordetella bronchiseptica* RB50^49^ in liquid broth media, prepare 5 mL of sterilized Miller’s LB broth (Fisher Scientific, Cat. #BP9723) in a 15 mL conical tube over a Bunsen burner flame, maintaining aseptic technique to prevent contamination. Stainer-Scholte media,^50^ which is specific for *Bordetella* spp., is also recommended.

**CRITICAL:** Use a culture medium that is suitable to the bacterial strain being used for this protocol, if it is not specifically *B. bronchiseptica*.

7. Using a 10 μL inoculation loop, isolate a single colony from a stock plate of *B. bronchiseptica* RB50 cultured on BGS agar and suspend into LB broth media. Stainer-Scholte medium can also be used but due to the complex chemical composition of the media that can impact batch effects and affect reproducibility, we recommend LB or even RPMI to perform the bacteria the overnight culture.
8. Incubate the liquid culture at 37°C, shaking at 200 rpm overnight (8–12 h) to allow for bacterial harvest in mid-to-late exponential growth phase (For *Bordetella* spp. we recommend OD_600_ 0.75–0.80).

***Note:*** When doing liquid cultures, leave the cap slightly open to allow for better oxygenation.

### Preparation of bacterial inoculums from bmEos challenge

**Timing: 30 min**

9. Measure the absorbance of each overnight culture using a spectrophotometer to determine bacterial growth at an absorbance of OD_600_.

**CRITICAL:** Do not use if the OD_600_ is ≥0.9–1 for *Bordetella* spp., as this indicates the culture has entered a stationary growth phase.

10. From this absorbance value, calculate the required volume to adjust the culture to 10^8^ CFU/mL by diluting the inoculum in PBS or culture media.

***Note:*** For *Bordetella* spp., an OD_600_ = 0.1 is equivalent to 10^8^ CFU, which is supported by previous literature^49^^,^^51^ and our previously published work.^1^ These calculations are specific for *Bordetella* spp. and if other bacteria are used, calculations will need to be adapted (see note below).

11. From the cell density of bmEos seeded into each well of the assay plate (generally 10^4-5^ bmEos per well), generate the bacterial inoculum required to add to each well at the desired multiplicity of infection (MOI). We recommend an MOI of 0.1 but if there is a need for higher MOI, we strongly recommend not to go over 10.
  a. Calculate the volume needed for dilution to achieve a concentration of bacteria of 10^8^, using the following formula: V_i_ x C_i_ = V_f_ x C_f_, where V_i_ is the starting volume and C_i_ is the initial concentration of the liquid culture. V_f_ and C_f_ are the desired volume and concentration.

***Note:*** Our *in vitro* work using bmEos^1^ was performed at 2 and 4 h post-infection. However, this may vary depending on experimental setting or the response desired for that assay (e.g., early vs. late eosinophilic responses to bacterial challenge).

12. To determine the final concentration of the inoculum, this will need to be plated onto BGS plates. Prepare at least three 10-fold serial dilutions from the inoculum added to each well, using sterile PBS as a diluent. (Figure 1).
  a. Plate each serial dilution^52^ of *Bordetella* spp. by spreading 100 μL of each sample on BG blood agar supplemented with 20 ng/mL of streptomycin.

**Figure 1:**
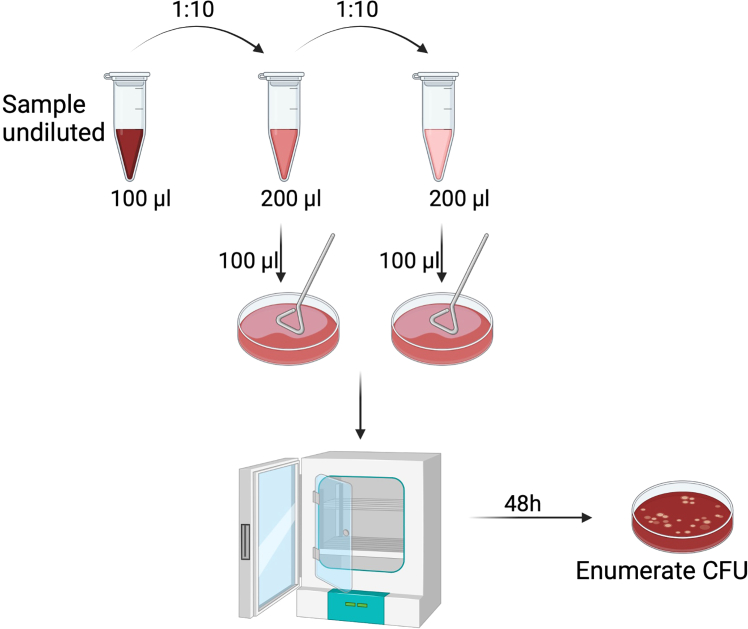
Enumeration of bacterial CFU following bmEos challenge Serial dilution schematic for enumerating bacterial CFU. At each time point, collect 100 μL sample and perform a series of 10-fold dilutions to achieve a countable CFU range between 20–200. Plate 100 μL of each diluted sample and incubate plates at 37°C for 48 h (adjust accordingly based on the bacterial species used). Upon formation of visible colonies from all plated samples, multiply by the respective dilution factor to enumerate CFU/mL. Created in BioRender. Gestal, M. (2025) https://BioRender.com/5dx5pvc.
13. Incubate each plated sample upside down (agar side up) at 37°C for 48 h, checking daily for contamination.
14. Enumerate the CFU of each plate, being sure to multiply the value by the dilution factor applied before plating the sample in order to calculate CFU/mL of the inoculum.
  ***Note:*** While we generally use assays without prior opsonization, if opsonization is required we recommend following these steps:
  a. Spin down the 0.1 OD_600_ inoculum and discard the supernatant.
  b. Resuspend in 0.5 ml of mouse serum for 1 h at 37°C at 200 rpm.
  c. After that time, spin down the inoculum and resuspend in Base Media #3 to get ready for the infections.

## Key resources table

**Table:** 

REAGENT or RESOURCE	SOURCE	IDENTIFIER
**Antibodies**		

Zombie Yellow fixable viability dye	BioLegend	Cat# 423103
Rat PE anti-mouse CD170 (Siglec-F), clone S17007L	BioLegend	Cat# 155505; RRID:AB_2750235
TruStain FcX plus (anti-mouse CD16/32), clone S17011E	BioLegend	Cat# 156603; RRID:AB_2783138
Rat AF488 anti-mouse/human CD11b, clone M1/70	BioLegend	Cat# 101219; RRID:AB_389305

**Bacterial and virus strains**		

*Bordetella bronchiseptica* RB50 strain	Cotter and Miller^49^	Cotter and Miller^49^

**Chemicals, peptides, and recombinant proteins**		

RPMI-1640, phenol-free	Gibco	Cat. #11835030
RPMI-1640	Gibco	Cat. #11875119
PBS, cell-culture grade	Thermo Fisher Scientific	Cat. #10010023
Heat-inactivated fetal bovine serum (FBS)	Gibco	Cat. #10437028
β-mercaptoethanol	Sigma-Aldrich	Cat. #60242
HEPES buffer solution (1 M)	Gibco	Cat. #15630080
L-glutamine (200 mM)	Gibco	Cat. #25030081
MEM non-essential amino acid solution (100×)	Gibco	Cat. #11140076
Sodium pyruvate (100 mM)	Gibco	Cat. #11360070
Bovine serum albumin (BSA) powder	Fisher Scientific	Cat. #BP9700100
Penicillin-streptomycin (10,000 IU/mL)	Gibco	Cat. #15140122
Phosphate-buffered saline (PBS)	Gibco	Cat. #10010049
Paraformaldehyde (PFA), granular	Electron Microscopy Sciences	Cat. #19210
LB broth media, Miller	Fisher Scientific	Cat. #BP1426500
Bordet-Gengou agar base	BD Life Sciences	Cat. #248200
Defibrinated sheep blood	VWR	Cat. #10145-964
Streptomycin	VWR	Cat. #45000-654
Glycerol	Thermo Fisher Scientific	Cat. #J61059.AP
Isoflurane inhalant anesthetic	Attane	Cat. #RXISO250
TRIzol reagent	Ambion Life Technologies	Cat. #15596018
RNaseZap decontamination solution	Invitrogen	Cat. #AM9782
EDTA, biotechnology grade (0.5 M)	VWR	Cat. #97062-836
Sodium azide	VWR	Cat. #97064-646
70% ethanol	VWR	Cat. #97064-768
Propidium iodide	Thermo Fisher Scientific	Cat. #P21493
SYTOX Orange	Thermo Fisher Scientific	Cat. #S34861
Trypan blue, sterile (0.4%)	VWR	Cat. #97063-702
Mouse recombinant FLT-3L (rmFLT-3L)	PeproTech	Cat. #250-31L
Mouse recombinant SCF (rmSCF)	PeproTech	Cat. #250-03
Mouse recombinant IL-5 (rmIL-5)	PeproTech	Cat. #215-15

**Critical commercial assays**		

PureLink RNA extraction kit	Invitrogen	Cat. #2365053
Luna one-step qRT-PCR kit	New England Biolabs	Cat. #E3005L
Thelper v.13 LEGENDplex chemokine ELISA	BioLegend	Cat. #740451
Thelper v.13 LEGENDplex cytokine ELISA	BioLegend	Cat. #741044
PureLink DNase set	Invitrogen	Cat. #12185010

**Experimental models: Cell lines**		

Murine bone marrow-derived eosinophils (bmEos)	This paper	Mai et al.^24^

**Experimental models: Organisms/strains**		

Mouse: BALB/cJ strain #000651	Jackson Laboratory	RRID:IMSR_JAX:000651
Mouse: C57BL/6J strain #000664	Jackson Laboratory	RRID:IMSR_JAX:000664

**Software and algorithms**		

LEGENDplex Data Analysis Software Suite	BioLegend	legendplex.qognit.com
GraphPad Prism (v.9.5.1)	GraphPad	graphpad.com

**Other**		

CFX Opus 96 real-time PCR system	Bio-Rad	Cat. #12011319
NovoCyte Quanteon flow cytometer	Agilent	Cat. #641181210464
NanoDrop OneC	Thermo Fisher Scientific	Cat. #840274200
Countess 3 automated cell counter	Invitrogen	Cat. #AMQAX2000
GENESYS 30 visible spectrophotometer	Thermo Fisher Scientific	Cat. #10861086
3D nutating mixer	VWR	76595-802
Mortar and pestle	Fisher Scientific	Cat. #S39830
Labconco BSL-2 biosafety cabinet	VWR	Cat. #76319-970
Eppendorf centrifuge, 5430R	Eppendorf	Cat. #97027-862
Vortex	VWR	Cat. #76416-196
New Brunswick Innova 40/40R incubated shaker	VWR	Cat. #75874-408
Eppendorf Easypet 3 electronic pipette controller	VWR	Cat. #10860-782
Sharp tip dissecting scissors (4^1^/_2_″)	VWR	Cat. #82027-578
Waugh forceps	VWR	Cat. #82027-428
Invitrogen Countess cell counting chamber slides and holder, disposable	Fisher Scientific	Cat. #C10228
Nalgene sterile disposable filter units	Thermo Fisher Scientific	Cat. #126-0020
70 μm cell strainer	Corning	Cat. #352350
UltraComp eBeads	Invitrogen	Cat. #01333341
Round-bottom polystyrene test tubes, 5 mL	Falcon	Cat. #352054
BD nonsterile syringes (5 mL)	VWR	Cat. #BD301027
Disposable serological pipettes (10 mL)	VWR	Cat. #75816-100
Disposable serological pipettes (25 mL)	VWR	Cat. #76320-280
Falcon centrifuge tubes, conical bottom (15 mL)	VWR	Cat. #21008-918
Falcon centrifuge tubes, conical bottom (50 mL)	VWR	Cat. #21008-940
Fisherbrand microcentrifuge tubes	Fisher Scientific	Cat. #14-666-318
Disposable Petri dishes (100 × 15 mm)	VWR	Cat. #470191-208
VWR disposable plastic transfer pipette, graduated	VWR	Cat. #10754-264
Gilson PIPETMANTM 4-pipette kit	Fisher Scientific	Cat. #F167360G
Fisherbrand pipette-specific tips 200	Fisher Scientific	Cat. #21-375D
Fisherbrand pipette-specific tips 1,000	Fisher Scientific	Cat. #21-375E
Nunc non-treated flasks	Thermo Fisher Scientific	Cat. #156800
Hard-Shell PCR 96-well plates	Bio-Rad	Cat. #HSP9601
Microseal-B PCR plate sealing film	Bio-Rad	Cat. #MSB1001
Corning CellBIND standard plates with lids	VWR	Cat. #66025-630
Tissue culture plates, non-treated, sterilized, non-pyrogenic	VWR	Cat. #10861-558

## Materials and equipment

**Table:** Bone marrow-derived eosinophil (bmEos) base media

Reagent	Volume	Final concentration
RPMI-1640 (phenol red-free)	367.3 mL	N/A
Heat-inactivated FBS	100 mL	20%
HEPES (1 M)	12.5 mL	25 mM
Penicillin-Streptomycin	5 mL	100 IU/mL penicillin, 10 μg/mL streptomycin
L-glutamine (200 mM)	5 mL	2 mM
Non-essential amino acids (100×)	5 mL	1×
Sodium pyruvate (100 mM)	5 mL	1 mM
β-mercaptoethanol	175 μL	50 μM
**Total volume**	**500 mL**	–

Store prepared base media at 4°C for up to 1 month. PMID: 33486726.^24^

**CRITICAL:** β-mercaptoethanol is very dangerous if not handled properly. It can be toxic if ingested and fatal if inhaled or absorbed through the skin. Inhalation of high concentrations can lead to central nervous system effects such as nausea, headache, dizziness, unconsciousness and coma. May cause respiratory tract irritation, and/or dyspnea (labored or difficulty breathing). Carefully confirm the concentration of β-mercaptoethanol is precisely 50 μM and not any higher, as this can be cytotoxic to the bmEos.

**Table:** Supplemented base media #1 (+rmFLT-3L, +rmSCF)

Reagent	Volume	Final concentration
bmEos Base Media	50 mL	N/A
Recombinant murine FLT-3L (rmFLT-3L), 10 μg/mL	500 μL	100 ng/mL
Recombinant murine SCF (rmSCF), 10 μg/mL	500 μL	100 ng/mL
**Total volume**	**50 mL**	–

Store supplemented base media at 4°C for up to 1 week.^24^

**Table:** Supplemented base media #2 (+rmIL-5)

Reagent	Volume	Final concentration
bmEos Base Media	50 mL	N/A
Recombinant murine IL-5 (rmIL-5), 5 μg/mL	100 μL	10 ng/mL
**Total volume**	**50 mL**	–

Store supplemented base media at 4°C for up to 1 week.^24^

**CRITICAL:** Always handle cell culture cytokines, reagents and media under a BSL-2 hood, using aseptic technique to prevent any contamination.

**Table:** FACS buffer

Reagent	Volume	Final concentration
PBS Cell culture grade	488 mL	N/A
Fetal Bovine Serum	10 mL	2%
0.5 M EDTA	2 mL	2 mM
Sodium Azide	250 μL (0.25 g)	0.05%
**Total volume**	**500 mL**	–

Store prepared FACS buffer at 4°C for up to 1 month.

**Table:** Bordet-Gengou with streptomycin (BGS) agar

Reagent	Volume	Final concentration
Bordet-Gengou agar base	500 mL	30 g/L
Glycerol, 100%	5 mL	10 g/L (1%)
Streptomycin	500 μL	20 ng/μL
Defibrinated sheep blood	75 mL	150 mL/L (15%)
**Total volume**	**580 mL**	–

Use agar immediately after preparation. Once the plates are cooled, they can be stored in the 4°C until utilization. Plates can be used for a maximum of a month after preparation unless they are dry before that.^1^^,^^53^

**Table:** Supplemented base media #3 (+propidium iodide, +HEPES)

Reagent	Volume	Final concentration
bmEos Base Media #2 (+rmIL-5)	15 mL	N/A
HEPES (stock = 1 M)	375 μL	25 mM
Sytox Orange Or Propidium iodide (stock = 5 mg/mL	10 μL	0.25 μM 3.3 μg/mL
**Total volume**	**15 mL**	–

Use prepared PI solution or Sytox immediately after preparation.

**CRITICAL:** You can do this assay selecting one of the options provided in the table; Propidium Iodine or Sytox. We recommend for personnel to start with Sytox as this is more stable and less toxic than PI, especially when the personnel is not highly trained on eosinophil *in vitro* manipulation.

## Step-by-step method details

### Collection of mouse bone marrow progenitor cells and bmEos differentiation

**Timing: 15 days**

***Note:*** This modified protocol is derived from Mai et al.^24^

1. Day 0:
  a. Place all materials required for cell isolation in a BSL-2 hood (Figure 2). Spray them with 70% ethanol and sterilize using UV treatment for 15 min.
    ***Note:*** If the laboratory has two BSL-2 hoods, we recommend using one for extraction and differentiation of bmEos and the other for bacterial infections.

**Figure 2:**
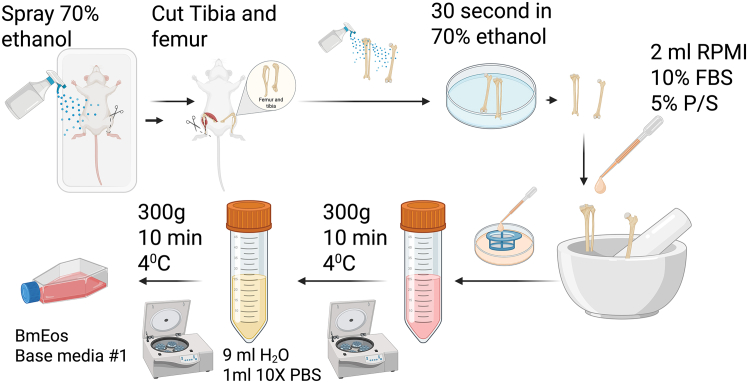
Isolation of bone marrow-derived progenitor cells Graphical schematic depicting the main steps required isolating murine progenitor cells from femur and tibia bone marrow. Created in BioRender. Gestal, M. (2025) https://BioRender.com/5dx5pvc.
  b. Euthanize mouse following IACUC guidelines and secure the mouse in a stretched position by pinning on a dissection platform in the cell culture hood.
  c. Spray mouse generously with 70% ethanol. Remove the skin of the lower half of the body to expose the major muscles.
    ***Note:*** Frequent soaking of the limbs in 70% ethanol can help soften the muscles and allow for easier removal from the fragile bones of the mouse.
  d. Sever each leg after reaching the connecting hip joint near the base of the lower limbs.
  e. Remove the major muscles from the femur and tibia, making sure not to break the bones.
    **CRITICAL:** The fibula, which is the smaller bone adjacent and connected to the tibia, may snap because it is extremely small and thin. However, it is more beneficial to take extra care not to break or snap the tibia or femur, as this will greatly affect cell yield, with the femur being the highest yielding bone being extracted in this procedure. If the femur breaks during any stage of this procedure, discard the sample and restart with another mouse.
  f. Place the bare bones into a sterile mortar containing approximately 1-2 mL of RPMI-1640 cell culture media, no Fetal bovine serum and no antibiotics added (herein refer as RPMI-Wash media).
  g. Applying stern pressure with an autoclaved pestle, grind the bones in the RPMI-Wash media using a circular motion to suspend the bone fragment evenly throughout the RPMI media.
    ***Note:*** The RPMI solution should now be cloudier with small but visible fragment particles in it. For target size reference of the desired bone fragments, they should not clog a P-1000 pipette tip.
    **CRITICAL:** Applying too much pressure or grinding up the bones for too long can decrease viability and overall cell yield of the extraction procedure.
  h. Pipette the homogenate solution through a 70–40 μm strainer while transferring to a petri dish. Wash the mortar 1-2 times with 1 mL of RPMI to ensure sufficient collection of the bone marrow.
  i. Use 1 mL of RPMI-Wash media to wash the filter and collect any remaining cells.
  j. Pipette the cell solution into a 50 mL conical tube, washing the bottom of the plate at least 3 times.
  k. Centrifuge each collection tube 300 × *g* for 10 min at 4°C.
  l. Carefully but swiftly discard the supernatant without disturbing the cell pellet.
    ***Note:*** Avoid pipetting to prevent shear stress and preserve cell viability.
  m. Lyse the red blood cells.
    i. Add 9 mL distilled water and quickly pipetting the pellet up and down twice.
    ii. Immediately add 1 mL 10× PBS to make solution isotonic and stop lysis.
    iii. Spin down 300 × *g* for 10 min at 4°C.
  n. Count the cells extracted from each mouse with a hemocytometer or automatic cell counter, using a 1:1 trypan blue solution. This will provide not only the cell number but also the viability which is indicative of quality.
    i. To prepare this mixture, add 10 μL of trypan blue and 10 μL of your suspension into a well of a 96-well plate.
    ii. Load 10 μL onto a Countess II automatic hemocytometer chip to quantify the cell concentration.
      ***Alternatives:*** Cells can also be counted manually using a traditional hemocytometer.
      ***Note:*** The cells will also need to be counted with the hemocytometer (manually or automatically) each time that the media is changed and prior to seeding the bmEos for each of the subsequent multi-well plate experiments.
  o. Resuspend cell pellet in 12 mL of Supplemented Base Media #1 (+rmFLT3-L, +rmSCF).
    ***Note:*** At this point we do not add the IL-5 supplementation yet, but mFLT3-L and rmSCF instead, to promote the proliferation and differentiation of mouse stem cells.^73^
  p. Adjust the volume accordingly to the acquired cell count of the sample to achieve a final concentration of 1 × 10^6^ cells/mL.
    ***Note:*** Typical yield at this point is around 18 × 10^6^ total cells.
  q. Transfer cell suspension into a T-75 (250 mL) cell culture flask. (Figure 2).
  r. Incubate at 37°C in 5% CO_2_ for 4 days, monitoring daily for contamination.
    ***Note:*** Mortar and pestles can be sterilized by spraying with 70% ethanol between mice. If the laboratory has two different cell incubators, we recommend using one for only cells and the other for the infection experiments. This minimizes undesired contaminations and keep physically separated clean from infected cell cultures.
2. Day 4:
  ***Note:*** When starting to work this day remember that eosinophils **DO NOT** need to be trypsinized. At the microscopy, eosinophils precursors appear large and they are mononuclear
  a. Pour all used cell culture media into a sterile 50 mL conical tube. Centrifuge cells at 300 × *g* at 4°C for 10 min (Figure 3).

**Figure 3:**
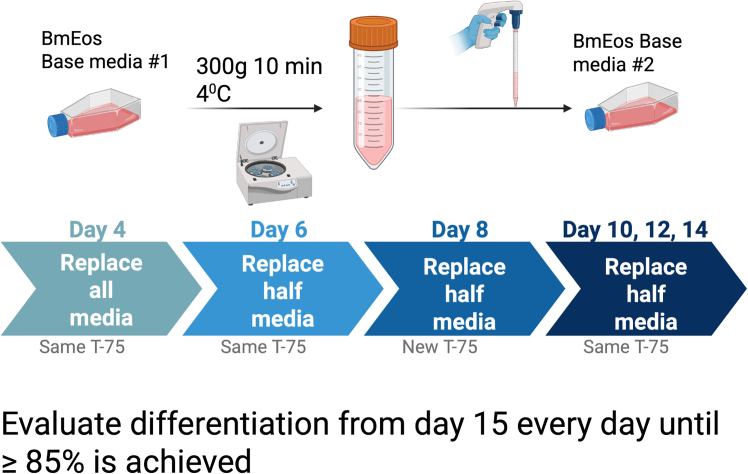
Differentiation of bone marrow-derived progenitor cells into eosinophils Graphical schematic illustrating the process of differentiating murine progenitor cells into bone marrow-derived eosinophils. Created in BioRender. Gestal, M. (2025) https://BioRender.com/5dx5pvc.
  b. Discard cell supernatant and resuspend pellet in 10 mL of Supplemented Base Media #2 (+rmIL-5).
  c. Transfer all contents to the **SAME** flask and proceed to use the remaining cells in the tube to count cells using the hemocytometer (**see Day 0, step “n”**).
  d. Incubate at 37°C in 5% CO_2_ for 2 days, monitoring daily for contamination.
3. Day 6:
  a. Pour all used cell culture media into a sterile 50 mL conical tube. Centrifuge cells at 300 × *g* at 4°C for 10 min.
  b. Pour out only **HALF** of the supernatant and resuspend cell pellet in Supplemented Base Media #2 (+rmIL-5).
  c. Transfer all contents to the **SAME** flask and proceed to use the remaining cells in the tube to count cells using the hemocytometer (**see Day 0, step “n”**).
  d. Incubate at 37°C in 5% CO_2_ for 2 days, monitoring daily for contamination.
4. Day 8:
  a. Pour all used cell culture media into a sterile 50 mL conical tube. Centrifuge cells at 300 × *g* at 4°C for 10 min.
  b. Pour out only **HALF** of the supernatant and resuspend cell pellet in Supplemented Base Media #2 (+rmIL-5).
  c. Transfer all contents to a **NEW** T-75 culture flask and proceed to count cells using the hemocytometer (**see Day 0, step "n"**).
  d. Incubate at 37°C in 5% CO_2_ for 2 days, monitoring daily for contamination.
5. Days 10, 12, and 14:
  a. Pour all used cell culture media into a sterile 50 mL conical tube. Centrifuge cells at 300 × *g* at 4°C for 10 min.
  b. Pour out only **HALF** of the supernatant and resuspend cell pellet in Supplemented Base Media #2 (+rmIL-5).
  c. Transfer all contents to the **SAME** flask and proceed to use the remaining cells in the tube to count cells using the hemocytometer (**see Day 0, step “n”**).
  d. Incubate at 37°C in 5% CO_2_ for 2 days, monitoring daily for contamination.

***Note:*** At day 14 evaluate differentiation as indication of quality (see next section). If differentiation is not ≥85%, then proceed to change half of the media and start incubation again for extra 2 days (repeat steps a-d).

**CRITICAL:** When the eosinophils are ready to be used, they will need to be used immediately. These cells are very delicate and cannot be frozen or stored.

### Analysis of bmEos quantity and purity: Flow cytometry for FACS sorting

**Timing: 2 h**

6. Prepare antibody solutions prior to performing flow cytometric analysis (Table 1).

**Table 1:** Antibody dilutions recommended for flow cytometry staining

Reagent	Dilution
Zombie Yellow Fixable Viability dye	1:500
Rat PE anti-mouse CD170 (Siglec-F)	1:200
TruStain FcX plus (anti-mouse CD16/32)	1:200
Rat AF488 anti-mouse/human CD11b	1:2000

PMID: 33486726.^24^

**CRITICAL:** Store prepared antibody solution in 4°C until utilization the same day. This has to be prepared fresh every time.

7. In a 15 mL conical test tube, resuspend fully differentiated bmEos (Day 15) in PBS to achieve a concentration of 10^6^ cells/mL.
8. Centrifuge at 300 × *g* for 5 min at 4°C, then swiftly remove the supernatant.
9. Incubate the cells with a live/dead stain for 30 min at 4°C in the dark.
10. Wash the cells with 3 mL of PBS and 0.1% BSA in each tube.
11. Centrifuge at 300 × *g* for 5 min at 4°C, then swiftly remove the supernatant.
12. Resuspend cells in 50 μL of anti-CD16/CD32 Fc blocking antibody solution (Table 1).
13. Incubate for 30 min at 27°C or room temperature.
14. Centrifuge at 300 × *g* for 5 min at 4°C, then swiftly remove the supernatant.
15. Add 50 μL of anti-SiglecF-PE in FACS buffer (Table 1).
16. Incubate for 30 min, without light, at 4°C.
17. Centrifuge at 300 × *g* for 5 min at 4°C, then wash with 100 μL of FACS buffer. Repeat centrifugation and washing.
18. Remove the supernatant and resuspend in 4% PFA-FACS solution.
19. Incubate for 1 h at 4°C without light.
20. Centrifuge at 300 × *g* for 5 min at 4°C, then wash with 100 μL of FACS buffer. Repeat centrifugation and washing.
21. Resuspend in 100 μL of FACS buffer for flow acquisition.

**Pause point:** Here, you may wish to stop and acquire the next day and store at 4°C overnight if the analysis is to happen at a later time point.

22. Acquire at least 15,000 positive events to determine the level of differentiation more rigorously but highly encourage to go to acquire at least 30,000 positive events.

**CRITICAL:** It is important that a minimum of 1.5 × 10^4^ positive events are collected for each sample, in addition to being live, CD11b^+^SiglecF^hi^ cells.

**Figure 4:**
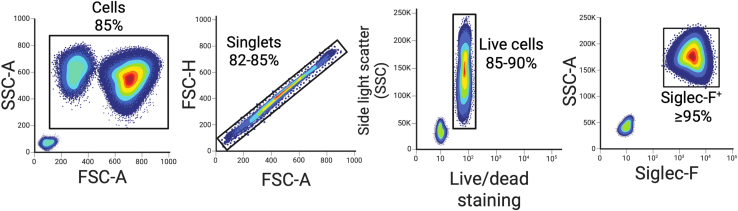
Flow cytometric gating strategy for assessment of eosinophil purity Schematic example of the flow cytometric gating strategy used to evaluate eosinophil quantity and purity, denoted with the expected percentages that were observed during our performed evaluations. Created in BioRender. Gestal, M. (2025) https://BioRender.com/5dx5pvc.

23. Confirm percentage of bmEos (CD11b^+^SiglecF^hi^) following the gating strategy shown in schematic (Figure 4).

**Figure 5:**
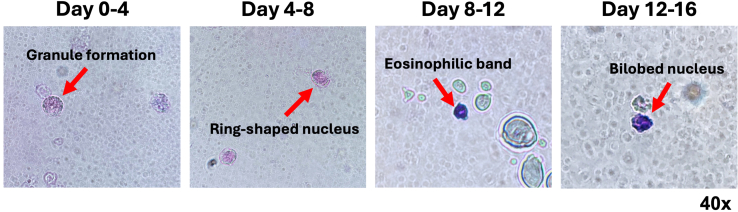
Staining of eosinophils along stages of differentiation Cell culture supernatants were collected at specific time points for cytospin, Wright-Giemsa staining, and light microscopy (original magnification, 40×) to assess key changes in eosinophil morphology along the differentiation process (indicated with red arrows). Please note there are some images containing air bubbles that formed when mounting the specimens onto microscope slides.

***Alternatives:*** Eosinophil differentiation and count can alternatively be determined via cytospin and Wright-Giemsa hematology staining for evaluation of eosinophilic cell morphology (Figure 5).

***Note:*** Following confirmation of purity, generally between day 15 and 17, bmEos are ready for use in functional assays and assessment following bacterial challenge. Differentiated bmEos no longer have a mononuclear morphology and decrease in size compared to precursor cells.

***Note:*** Bone marrow-derived eosinophils are terminally differentiated cells and do not proliferate, so therefore the generated cultures cannot be passaged. We recommend using differentiated bmEos on or before day 17-18 to maintain full viability.

### Functional analysis of bmEos: Cytotoxicity assay

**Timing: 10 h**

The goal of this experiment if to determine the rate of eosinophil death upon encountering with bacteria. This experiment is designed to evaluate eosinophil death in a dynamic, temporal manner. The strength of this assay lies in its adaptability. By simply changing the reagent used you can mechanistically investigate the pathway driving cell death (for example using probes for caspase-1 activation or generally assessing death with reagents such as Annexin V). This experiment is performed in a TECAN like platform that allow for the evaluation of positive signal as a correlative indication of eosinophil death.

24. Pour the bmEos supernatant from a T-75 culture flask into a 15 mL conical tube and centrifuge the cell solution at 300 × *g* for 10 min.
25. Discard the supernatant and resuspend the pellet in 15 mL of Supplemented Base Media #3 (+rmIL-5, +PI, +HEPES).

**CRITICAL:** The media used in this assay must be phenol-red free. The plate should be a black plate to avoid bleeding of positive signal from one well to another.

26. Load bmEos into a 96-well plate (Figure 6).

**Figure 6:**
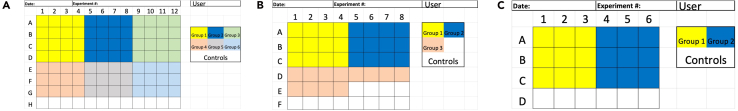
Multi-well plate experimental layouts Recommended layouts for 96-well (A), 48-well (B), and 24-well (C) plates for use in bmEos assays. Each color represents a different sample groups which are denoted for each plate layout, with white being blank controls. Columns are used for technical replicates, and each row indicates a different biological replicate. Each experiment should be repeated at least 3 independent times, using the same number of technical and biological replicates. Created in BioRender. Gestal, M. (2025) https://BioRender.com/5dx5pvc.
27. Add the prepared bacterial inoculums and centrifuge the plate at 300 × *g* for 5 min.

**CRITICAL:** For all performed assays described in detail below, control groups should include eosinophils unchallenged and bacteria alone. We also recommend including positive control groups, such as eosinophils treated with 20 ng/mL of LPS and/or 1% Triton-X-100. At each time, plate for bacterial counts two wells of each control to identify any possible contamination. Each time the experiment is done it should contain at least 3 technical replicates per time and condition. We recommend to also do it at least 4 or more times independently to obtain sufficient number of biological replicates that the results are robust to determine differences between treatments.

28. Load the plate into the TECAN Spark microplate reader at 37°C in 5% CO_2_.
29. Allow the assay to run overnight (≥ 10 h), taking measurements every 30 min.

***Alternatives:*** Other modes of assessing cytotoxicity can be used, such as detection of Annexin-V, ApoTracker probes, Sytox green, or caspase activation. If this is the case, adjust reagents accordingly.

### Functional analysis of bmEos: Bactericidal and internalization assays

**Timing: 10 h**

The goal of this experiment if to determine the ability of eosinophils to kill bacteria. We propose to perform this assay as a temporal investigation, by taking measurements of bacterial CFU at different time points. In this assay, samples of the bacteria in co-culture with eosinophils are plated for bacterial CFU at different time points, which will indicate bacterial survival or killing by eosinophils.

30. After loading bmEos onto a 96-well plate and addition of prepared bacterial inoculums (see materials and equipment), centrifuge the plate at 300 × *g* for 5 min.

***Note:*** A MOI of 0.01–0.1 is the best MOI to perform phagocytic assays for *Bordetella*. Using any MOI that exceeds 10 is NOT recommended, as this induces rapid cytotoxicity that limits the resolution of this assay.

31. Collect the samples from the wells designated for time 0 in an Eppendorf tube. Place the plate back in the incubator until the next sampling time.

**CRITICAL:** Plates, liquid media, and sterile PBS should be prepared before starting the assay.

32. Vortex the acquired time-point samples. Perform a series of 10-fold dilutions in sterile PBS (Figure 1) and plate 100 μL of each diluted sample onto BGS agar plates.

***Note:*** We recommend having one row of the plate for only eosinophils that can be plated at 0 dilution at all sampled time points. This is to guarantee that no contaminations occur during the experiment.

33. Repeat sample acquisition and plating for the assigned wells of 30 min, 2 h, 4 h, and 8 h post-infection.

***Alternatives:*** The sampling times can be changed according with previous results and experimental setup. However, it is important to plate several dilutions the first time to ensure at least one plate that is in countable range (2-400 CFU).

34. Incubate each BGS plate for 48 h at 37°C.
35. Record the counted CFU from each plate and multiply enumerations by the appropriate dilution factors to achieve total CFU/mL.
36. Graph the calculated CFU values using time on the x-axis and Log_10_ CFU/mL on the y-axis.

***Alternatives:*** Modifications of this assay can include collection of RNA for qRT-PCR analysis of eosinophil transcriptional responses at different times, collection of supernatants for evaluation of cytokines and chemokines (see below), or flow cytometry assays of labeled bacteria to evaluate internalization.

### Functional analysis of bmEos: Multiplex ELISA

**Timing: 5–7 h**

The goal of this experiment if to determine the specific and unique cytokine/chemokine responses of eosinophil to different stimuli. In this assay, supernatant of eosinophils in co-culture with bacteria will be used to investigate the cytokines/chemokines secreted in respond to specific stimuli. For this experiment we use supernatant of eosinophils challenged with bacteria (in our case *Bordetella* spp.) for example bactericidal or internalization assays.

***Note:*** This protocol is a simplified version for LEGENDplex multiplex ELISA kits provided by BioLegend. We used the mouse proinflammatory chemokine and mouse T helper (Th) cytokine panels. Before using this protocol, adjust it as recommended by the vendor as necessary.

37. Prepare a 96-well plate, making sure to assign two columns for standards. Homogenize the pre-mixed beads by vortexing for at least 1 min.

***Note:*** All samples should be run in at least duplicate to account for technical errors.

38. Dilute 25 μL of the wash buffer in 475 μL of deionized water (dH_2_O).
39. Prepare the standard stock solution (C7) and incubate at room temperature for 10 min. Add 75 μL of assay buffer into tubes for C6-C1 standard dilutions. Perform serial dilutions by adding 25 μL of the previous standard solution to the next tube, until all standard concentrations have been diluted, being sure to vortex between each tube preparation.
40. Add 25 μL of assay buffer, 25 μL of standard solution, and 25 μL of bead solution in each designated well.

**CRITICAL:** This is the most important step in the ELISA. Vortex prior to making new dilutions and vortex prior loading the plate. If the standard curve is not good the whole assay won’t be meaningful and will need to be disregarded.

41. For extracted bmEos cell supernatants, add 25 μL of assay buffer, 25 μL of sample, and 25 μL bead solution to each well to achieve a final volume of 75 μL per well.
42. Seal the plate; cover the plate and lid completely to protect the plate from light.

**CRITICAL:** The plate should always be on top of a lid or something else that lift it from all surfaces.

43. Shake at 800 rpm using a rocking platform for 2 h at 27°C. Centrifuge plate at 250 × *g* for 5 min.
44. Replace the supernatant with 200 μL of wash buffer. Incubate covered plate for 1 min covered with the foil to protect from the light. Centrifuge at 250 × *g* for 5 min and remove the supernatant.
45. Add 200 μL of wash buffer and pipette to mix; cover from light and incubate for 1 min. Centrifuge the plate at 250 × *g* for 5 min; replace supernatant with 25 μL of detection antibody. Seal the plate and cover plate and lid completely.
46. Shake at 800 rpm for 1 h at 27°C. Directly add 25 μL of Streptavidin-Phycoerythrin (SA-PE) and **do not mix.** Seal and cover the plate.
47. Shake at 800 rpm for 30 min at 27°C.

**CRITICAL:** Do not mix after adding the SA-PE.

48. Spin down the plate at 250 × *g* for 5 min. Discard the supernatant and mix 200 μL of wash buffer. Cover with aluminum foil and incubate for 1 min.
49. Repeat centrifugation and washing. Resuspend in 150 μL of wash buffer.
50. Acquire ELISA data from the flow cytometer.

## Expected outcomes

### bmEos differentiation

This process generally commences with 18 × 10^6^ progenitor cells isolated from the bone marrow of BALB/c mice. While differentiation process occurs, number of cells decreases and by day 14 approximately 95% of the cells are differentiated eosinophils (approx. 10 × 10^6^ cells total). If the mice background is C57BL/6J mice, the number of differentiated eosinophils is lower (about half less or even less if the operator is not proficient with the technique). Moreover, differentiation of C57BL/6J will need up to day 15-16 as these cells do not respond as well to the IL-5 stimulation. These counts are based on our experience, but number can vastly vary according to the operator experience, changes in media which can lead to cell loss, and precision in counts while performing the cell differentiation (Figure 3). It is important to note that the final eosinophil yield and differentiation rate varied between different mouse strains and that is why it is extremely important to always have uninfected controls in all your experiments. This will allow you to evaluate interexperiment reproducibility strengthening your results and rigor.

### bmEos cytotoxicity assay

The expectation is that the negative control will have a mortality rate below 5%, and the positive control will not exceed 90% mortality by the end of the experiment. If mortality in the negative control exceeds 10% before the experiment concludes, this will indicate a problem, and the experiment will need to be discarded. If the positive control reaches a plateau in OD_600_ at early time points, the analysis will only include time points prior to the plateau, as this would otherwise compromise the measurable range of the experiment.

**Figure 7:**
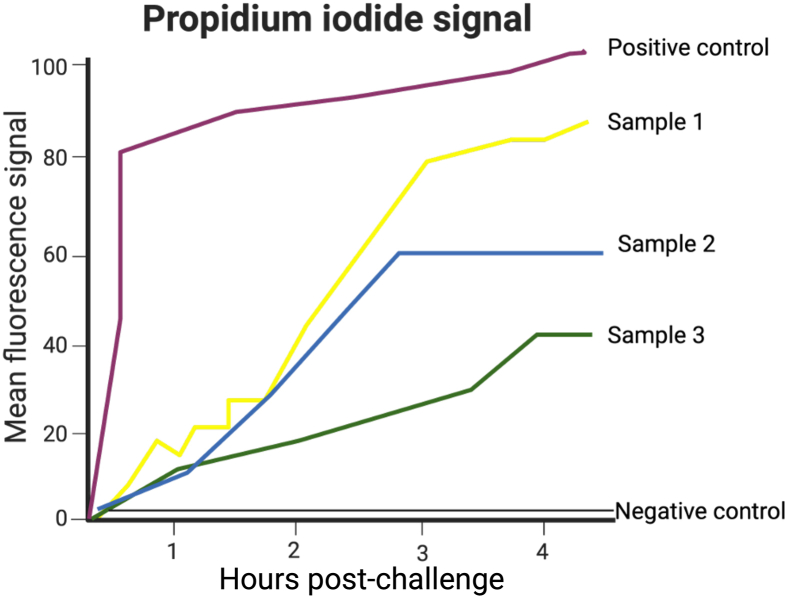
Expected results of dynamic cytotoxicity assay Schematic predicting the expected results of dynamic cytotoxicity assays using bmEos. The black multi-well plate is positioned into a temperature-regulated plate reader connected to CO_2_ and read at 605 nm for propidium iodide signal, which is directly proportional to cytotoxicity. The instrument should be connected to with the temperature regulated for overnight incubation at 37°C. The program should take measurements every 10 min for 10-12 h (we recommend acquiring data overnight), with each reading measuring 6 different areas of the well. Parameters can be changed accordingly to dye, bacteria or desired time points post-infection. Created in BioRender. Gestal, M. (2025) https://BioRender.com/5dx5pvc.

When evaluating the results of this experiment it is important to first analyze the controls. It is expected that eosinophils alone, negative control, do not present a death rate over 5% by the end of the experiment. Our positive controls, LPS, Triton-X-100 or other Toll-like receptor agonists, will promote death at different levels. We have seen that Triton can cause rapid cell death, and LPS, while also causes death, cells die increasingly with the time, providing a better control for the dynamic evaluation of death over time. Prior to analysis, we graph the average of the 2-4 technical replicates along the x-axis vs. time and in the Y-axis the average death corresponding to that time and infection group (Figure 7).

We recommend the use of Sytox at the beginning to get familiar with the assay, because it does not cause cell death and makes it easier for the personnel that is not highly proficient with the usage of eosinophils *in vitro*. Furthermore, Propidium iodide can interfere with eosinophil activation status, which has an effect of our indirect evaluation of death. If three biological replicates continue to offer inconsistent results. While experienced personnel can obtain reliable results with PI, we recommend using Annexin V, Sytox, or Caspase-1 (See key resources table) using the same approach and adapting the concentrations of each reagent according to manufacturer’s recommendations. Results with any of these recommended reagents will be similar to those shown in Figure 6.

When we use *Bordetella* spp., eosinophil death along 8 hours reaches a maximum of 80% at an MOI of 1. We do not recommend MOI of 10 because then eosinophil mortality will occur too fast to be able to dissect differences between strains, conditions, or inoculums.

### bmEos bactericidal and internalization assays

**Figure 8:**
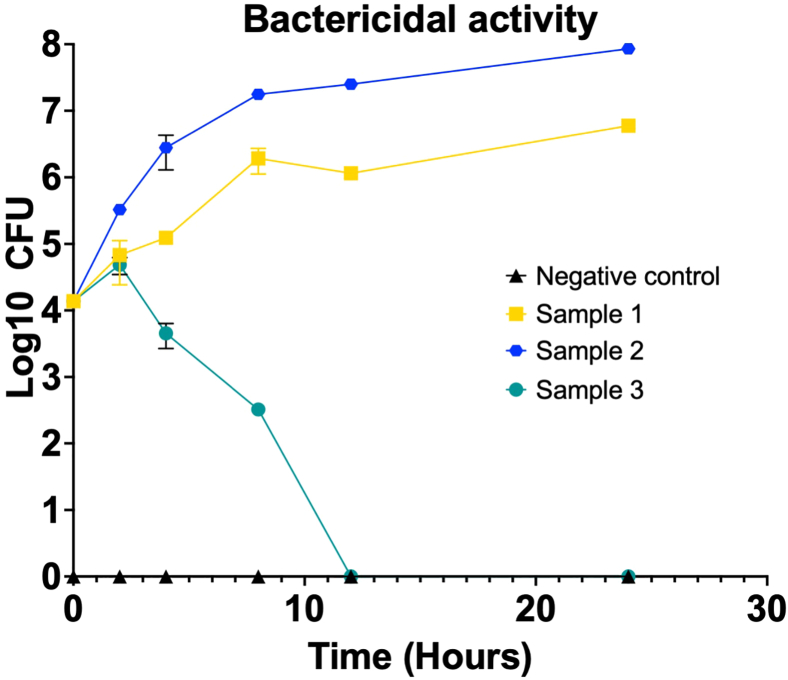
Expected results of bactericidal activity bmEos assay At different times, enumerate the recovered CFU along selected incubation times, and graph the log_10_ CFU/mL (y-axis) vs. hours post-infection (x-axis). Negative controls should be also plated at each time point as a control for contamination. Each symbol represents the mean ± SEM for at least 3 biological replicates, for each time point and each experimental setting. Statistics are not shown, but Tukey's Two-Way ANOVA with multiple comparisons is recommended. Created in BioRender. Gestal, M. (2025) https://BioRender.com/5dx5pvc.

It is anticipated that the bactericidal activity will show increasing levels of bacterial death across time. For example, starting with an MOI of 0.1 (e.g. 10^3^ CFU), a steady decrease in CFU is expected throughout the experimental time course that can reach even undetectable growth (that is why it is important to plate also undiluted sample). We also anticipate that bacteria cultured alone will exhibit growth at the different time points. However, bacterial replication in eosinophil media (Supplemented Base Media #2) may differ from standard conditions and could affect replication rates. We recommend calculating the bacterial replication rate^53^ in this medium to validate assay conditions. If the bacteria alone in the media present decrease CFU a long time, suggestive of bacterial killing, then the assay needs to be adapted and calculations to normalize the bacterial death with and without eosinophils will need to be applied. For CFU enumeration, all plates within a CFU range of 20-200 should be counted. It is recommended to count at least two dilutions per sample and time point for greater accuracy and increased rigor. Data are visualized using time on the x-axis vs. Log_10_ CFU/mL on the Y-axis (Figure 8). If duplication media is calculated, represent the results as type of media on the X-axis and time in the Y-axis.

In our assays, wildtype *Bordetella* bronchiseptica at an MOI of 0.1 is all killed by 8 h post-infection, however, other mutants are completely killed by 4 h. Contrary using MOI of 10 will lead to eosinophil death and bacterial growth in the media.

### bmEos multiplex ELISA

**Figure 9:**
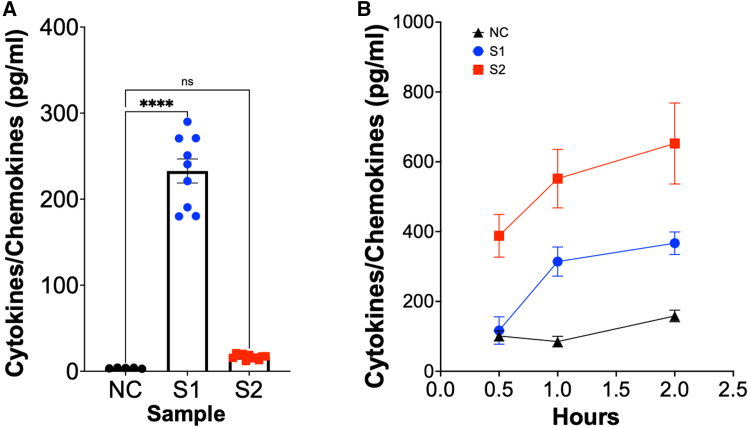
Expected results for bmEos multiplex ELISA Black symbols represent the negative control group, blue represents the unknown sample group, and red represents the known positive control. (A) Column representation of example data for that represents expected results of one specific target from the multiplex panel, where NC stands for negative control, S1 is unknown sample 1, and PC is positive control. Tukey's One-Way ANOVA with multiple comparisons was performed (ns = non-significant, ∗∗∗∗ = p < 0.0001). (B) An XY graph that represents the concentration (pg/mL) of a single cytokine/chemokine target from the multiplex panel over a time course of infection. Each symbol represents the mean ± SEM of at least 3 biological replicates for each time point and each experimental setting. Statistics are not shown, but we recommend using Tukey's Two-Way ANOVA with multiple comparisons.

This data will reflect the concentration of different cytokines/chemokines in pg/mL. The working range will vary specifically for each cytokine or chemokine studied. In our studies, we used several kits, being one example the mouse proinflammatory chemokine panel (Cat. #740451). Once the concentration of protein was calculated on the cloud-based software, all data was exported as an excel file and graphed using GraphPad. Columns were used when we only used to one time point, and the results were presented as columns. However, when we evaluated the response over time, grouped was the format selected, and the results were presented using time on the X-axis vs. concentration of the cytokine on the Y-axis (Figure 9). We recommend that changes in cytokines/chemokines concentrations are also evaluated by performing targeted ELISA kits for each molecule of interest. This will increase robustness showing the reproducibility of the data using two different methods. Single ELISA assays can also be used, and in fact, are recommended to evaluate the concentration of targeted specific cytokines. In this case, please follow the manufacturer’s recommendations.

Expect low concentrations of cytokines when doing the assay as the number of cells is 10^4-5^ and it will be diluted in 100 μL of volume used to start the assay.

## Quantification and statistical analysis

### bmEos cytotoxicity assay

Statistical analysis must contain at least 4 biological replicates including 3-4 technical replicates each. However, based on our experience it is better to have at least 5-6 biological replicates for more rigor and reproducibility. These results do not follow a normal distribution; however, a test such as Kolmogorov-Smirnov, can be used to evaluate normality. For the analysis, the average of the technical replicates will be utilized as sample 1, and so on. There will always be at least three groups (negative control, at least one positive control or more, and the sample group that can be one or more), this means that Two-Way ANOVA is recommended. If standard deviation is over 20%, at least eight biological replicates are recommended.

It is important to do several death/viability assays to corroborate the results. We recommend combining PI and Annexin-V detection methods to increase replicability and show with alternative methods the same outcome.

### bmEos bactericidal and internalization assays

It is important to always confirm that the negative controls have work appropriately and that no bacteria grow on the negative wells along the whole course of the experiment. The presence of bacterial colonies in the negative control samples (eosinophils alone) would indicate contamination, and the entire assay should be discarded. Once controls have been validated and confirmed for analysis, record the raw counts in an Excel spreadsheet and perform all necessary calculations to correct for the dilution factor used in each sample (Figure 1). Total CFU/mL (after dilution correction) are graphed in GraphPad as grouped data, with variables including bacterial strain and time.

This assay must contain at least 4 biological replicates including 3 or more technical replicates each. For the analysis the average of the technical replicates will be utilized as sample 1, and so on. These results do not follow a normal distribution; however, a test such as Kolmogorov-Smirnov, can be used to evaluate normality. There will always be at least three groups (negative control, at least one positive control or more, and the sample group that can be one or more), this means that Two-Way ANOVA is recommended. If standard deviation is over 20%, at least six biological replicates are recommended.

### bmEos multiplex ELISA

For data analysis, we use Qognit, the cloud-based LEGENDplex analysis software provided by BioLegend We advise taking advantage of the procedure tutorials also provided by BioLegend and contact the technical support when required. We analyzed the data by uploading the .fcs files from the flow cytometry instrument to the software and followed the instructions as provided in the BioLegend software manual. For more details, please contact BioLegend technical support. In each analysis, include the positive and negative controls and use as base line the concentration obtained from the eosinophils unchallenged sample. As there are two technical replicates, graph the average.

This ELISA must contain at least 4 biological replicates including 2 or more technical replicates each. For the analysis the average of the technical replicates will be utilized as sample 1, and so on. There will always be at least three groups (negative control, at least one positive control or more, and the sample group that can be one or more). These results do not follow a normal distribution; however, a test such as Kolmogorov-Smirnov, can be used to evaluate normality. If data is evaluated as column, then t-Test will be used if only two samples are included in the analysis. However, most likely 3 groups (including the controls) will be part of the analysis and One-Way ANOVA is recommended. When time responses are being evaluated the analysis includes two variables, time and concentration, which means that Two-Way ANOVA is recommended. If the standard deviation is over 20%, more biological replicates are recommended. In our hands we have done 6 or more biological replicates to compensate the deviation of the data.

## Limitations

### bmEos differentiation

Bone marrow-derived eosinophils are terminal cells and cannot be passaged, have no replication abilities, nor can they be stored. The *in vitro* experiment should be concluded without any passages, when the differentiation rate is ≥85% (preferably higher). Once eosinophils are differentiated need to be used as soon as possible, preferably within a day, before they start to die.

Another limitation is that this assay will not permit to differentiate between circulatory and tissue resident eosinophils. Similarly, expression of other markers might also be affected for the *in vitro* conditions.

### bmEos cytotoxicity assay

Small differences in MOI can significantly affect the speed at which eosinophils die. Similarly small changes in temperature, humidity or other external cues. Due to the high sensitivity of these cells to die we recommend doing this assay a minimum of 6 times if not more. It is simple enough to have enough number of replicates that allows you to compensate the variability of the data generated by the uncontrollable effects on eosinophil death of external environmental stressors. While Sytox death evaluation increases in a timely manner, Propidium iodide can cause cell death so we expect to see how the cells eventually die but this should not be rapid nor pronounced. Because of the high sensitivity of propidium iodine and the facility of eosinophils to die, if after three biological replicates the results are inconclusive, we recommend using other dynamic death assay such as Sytox or Annexin V (See key resources table).

### bmEos bactericidal and internalization assays

Very low MOI are recommended because addition of gentamycin affects the outcome of the experiment. This means that sometimes it might be difficult to hit the appropriate MOI. When this happens the results will show differences in bacterial death up to a certain point from which all eosinophils are death and bacteria is just growing on the eosinophils media. If this happens, repeat again at lower MOI. As antibiotic is not recommended for this assay, working in sterile conditions is highly necessary to prevent false results due to external contaminations, and only highly trained personnel should be doing this assay to obtain reliable data.

### bmEos multiplex ELISA

Using the multiplex generally provides very good results. However, it is recommended that only trained personnel perform this assay as in each washed beads can be lost affecting the overall results obtained. As the number of eosinophils per well is generally low, the concentration of cytokines might fall under the limit of detection, if that is the case for multiple cytokines of the assay, it is best to disregard that particular assay and start from the beginning using higher concentrating eosinophils in the assay. This is necessary to avoid misinterpretation of data due to the lack low concentration of cytokines/chemokines on the sample.

Overall, obtained results cannot be used to compare phenotype subsets bmEos versus isolated from tissues (e.g., lungs). Utilizing murine models of eosinophils *in vivo* must be done to make conclusions about physiological or pathological responses following infection. However, these *in vitro* experiments can provide foundational evidence for the formation of hypotheses about what occurs at the organism/organ/tissue-specific levels and how eosinophils may be driving these outcomes, as well as what may be key determinants in these pathways/processes.

## Troubleshooting

### Problem 1

• Low cell count/yield or low viability of bmEos (see “Analysis of bmEos quantity and purity: Flow cytometry for FACS sorting” step).
• Sometimes by the time that the assay can be perform the total count of eosinophils is very low. When this happens is recommended to use these eosinophils for a small pilot experiment reducing the number of samples and technical replicates but always including the appropriate controls. However, to avoid that this from happening again there are some potential solutions.

### Potential solution

• Carefully confirm the concentration of β-mercaptoethanol is precisely 50 μM and not any higher, as this can be cytotoxic to the bmEos.
• Prepare fresh cell culture base media and cytokine cocktails. We recommend plating a small aliquot as a quality control that no contaminations are found on any of the components of the media, neither the media.
• Make sure that the FBS being used has been heat-inactivated (56°C for 60 min)
• Ensure cell concentration is no less than 10^5^ cells/mL but not over than 10^6^ cells/mL. If so, seed cell suspension in a smaller culture container.
• Ensure the concentration is not much higher than 10^6^ as it can affect survival of eosinophils.
• If issues continue to arise, please refer to other more detailed protocols focused on isolation and culture of mouse and human eosinophils.^24^^,^^54^

### Problem 2

• Spontaneous cell death during differentiation or during the eosinophil death assay (see “Functional analysis of bmEos: Cytotoxicity assay” step).
• Eosinophils are very sensitive cells and spontaneous death, or degranulation can happen if conditions are not optimal. If this occurs, those eosinophils should be disregarded. However, moving forward there are some potential solutions.

### Potential solution

• Discard the media and prepare a new one with newly dissolved cytokines.
• Confirm the concentration of β-mercaptoethanol. This is a highly cytotoxic agent that if added in excess can led to cell death.
• Confirm that the incubator is at 37°C and the level of CO_2_ is 5%.
• Make sure that your media is not contaminated (it is recommended to inoculate some media into LB liquid media from time to time to ensure is not contaminated)
• Make sure that the concentration of propidium iodine is not higher than recommended in this protocol, it can cause spontaneous death.
• If the inoculated samples (combined with bacteria) die too fast the inoculum might be too high, dilute more the inoculum next time.
• During the death assay it could be that the concentration of bacteria is too high. Make sure your spectrophotometer is in good shape prior to the assay. Double check the calculations and make sure that the MOI is appropriate. It is recommended to perform a pilot experiment evaluating different MOIs to select those that allow for a better working range.

### Problem 3

• Bacteria are uncountable/undetectable during the sampling (see “Functional analysis of bmEos: Bactericidal and internalization assays” step).
• Due to the low MOI that is required for these assays sometimes the final inoculum might be too low.

### Potential solution

• It is important to make sure that bacteria are able to grow or at least not die in the eosinophil free wells, as a control. If bacteria are undetectable, try to increase the MOI.
• If bacteria die in the eosinophil-free wells, then prepare fresh media with newly made cytokines, as those might impair bacterial growth/survival.
• Double check the calculations to ensure the MOI is correct.

### Problem 4

• Contaminations during the internalization assay (see “Functional analysis of bmEos: Bactericidal and internalization assays” step).

### Potential solution

• If there is bacterial grow in the eosinophil’s alone wells, this can indicate that the media is contaminated. Prepare fresh media.
• If there is growth of two different bacteria in the bacteria alone wells (our bacteria of interest and another morphotype), this can indicate that the inoculum was contaminated. If this is the case, prepare new bacterial culturing media to prevent contaminations.
• Any type of contamination can indicate that the operator is not working in sterile conditions. Then it is recommended that an experience professional does the assay as a control that the contamination source was the operator. If that is the case, retrain the operator in sterile techniques.

### Problem 5

• No conclusive results from the ELISA due to increase variability (see “Functional analysis of bmEos: Multiplex ELISA” step).
• This can be an issue due to the low concentration of bmEos per well. However, there are several solutions to prevent this from happening.

### Potential solution

• Concentrate the number of bmEos per well to 10^5^ or just below 10^6^. This will also prevent the high variability that can result from low concentrations of protein in the supernatant.
• Increase the number of biological replicates. This will provide more robustness, and it is a good practice to always have high numbers of replicates when working with eosinophils *in vitro.*

## Resource availability

### Lead contact

Further information and requests for resources and reagents should be directed to and will be fulfilled by the lead contact, Dr. Monica Cartelle Gestal (monica.cartellegestal@lsuhs.edu or mcarges@gmail.com).

### Technical contact

Technical questions on executing this protocol should be directed to and will be answered by the technical contact, Katelyn M. Parrish (katelyn.parrish@lsuhs.edu) as well as the lead contact, Dr. Monica Cartelle Gestal (monica.cartellegestal@lsuhs.edu or mcarges@gmail.com).

### Materials availability

This study did not generate any new reagents or materials.

### Data and code availability

This study did not generate any new data or code.

## Acknowledgments

We would like to acknowledge the support of the funding bodies, which had no role in the study design, data collection, data analysis, or data interpretation: 10.13039/100000002NIH/10.13039/100000057NIGMS Centers of Biomedical Research Excellence (COBRE) grant award (P20GM134974), Center of Excellence for Arthritis and Rheumatology Intramural Award, Intramural Research Council Seed, and Start-up Package from 10.13039/100010519LSU Health Sciences Center at Shreveport.

We would like to acknowledge the core facilities associated with the COBRE, the CAIPP Bioinformatics and Modeling Core (RRID: SCR_024779), and the CAIPP Immunophenotyping core (RRID: SCR_024781).

We would like to also acknowledge the Multidisciplinary Training in Cardiovascular Pathophysiology (MTCP) Predoctoral T32 NIH Fellowship (5T32HL155022-03) of K.M.P., provided by the Center for Cardiovascular Diseases and Sciences (CCDS) at LSU Health Sciences Center at Shreveport. We would like to acknowledge the Ken Peterson Memorial Predoctoral Fellowship of K.M.P., provided by the Department of Microbiology and Immunology of LSU Health Sciences Center at Shreveport.

We would like to acknowledge Sanna Younger from BioLegend and Revvity, for the support and continued guidance on all aspects for performing the LEGENDplex ELISA.

All schematic figures included in this protocol created with BioRender are cited in their respective figure legends.

We would like to acknowledge Dr. Helene Rosenberg who provided us with support, guidance, and protocols while we were starting our eosinophil adventure.

## Author contributions

K.M.P.: optimizing protocols, performing the assays, analyzing the results, writing, and editing. T.L.W.: optimizing eosinophil isolation and culture and protocols and editing. M.C.G.: optimizing eosinophil isolation, culture, differentiation and protocols, writing and editing, funding, project administration, and supervision.

## Declaration of interests

The authors declare no competing interests.

## References

[1] First N.J., Parrish K.M., Martínez-Pérez A., González-Fernández Á., Bharrhan S., Woolard M., McLachlan J.B., Scott R.S., Wang J., Gestal M.C. (2023). Bordetella spp. block eosinophil recruitment to suppress the generation of early mucosal protection. Cell Rep..

[2] Tiwary M., Rooney R.J., Liedmann S., LeMessurier K.S., Samarasinghe A.E. (2021). Eosinophil Responses at the Airway Epithelial Barrier during the Early Phase of Influenza a Virus Infection in C57BL/6 Mice. Cells.

[3] LeMessurier K.S., Rooney R., Ghoneim H.E., Liu B., Li K., Smallwood H.S., Samarasinghe A.E. (2020). Influenza A virus directly modulates mouse eosinophil responses. J. Leukoc. Biol..

[4] Samarasinghe A.E., Melo R.C.N., Duan S., LeMessurier K.S., Liedmann S., Surman S.L., Lee J.J., Hurwitz J.L., Thomas P.G., McCullers J.A. (2017). Eosinophils Promote Antiviral Immunity in Mice Infected with Influenza A Virus. J. Immunol..

[5] Eddens T., Elsegeiny W., Nelson M.P., Horne W., Campfield B.T., Steele C., Kolls J.K. (2015). Eosinophils Contribute to Early Clearance of Pneumocystis murina Infection. J. Immunol..

[6] Figueiredo R.T., Neves J.S. (2018). Eosinophils in fungal diseases: An overview. J. Leukoc. Biol..

[7] Muniz V.S., Silva J.C., Braga Y.A.V., Melo R.C.N., Ueki S., Takeda M., Hebisawa A., Asano K., Figueiredo R.T., Neves J.S. (2018). Eosinophils release extracellular DNA traps in response to Aspergillus fumigatus. J. Allergy Clin. Immunol..

[8] Cottin V. (2016). Eosinophilic Lung Diseases. Clin. Chest Med..

[9] Garro A.P., Chiapello L.S., Baronetti J.L., Masih D.T. (2011). Eosinophils elicit proliferation of naive and fungal-specific cells in vivo so enhancing a T helper type 1 cytokine profile in favour of a protective immune response against Cryptococcus neoformans infection. Immunology.

[10] Wagner J.M., Franco M., Kephart G.M., Gleich G.J. (1998). Localization of eosinophil granule major basic protein in paracoccidioidomycosis lesions. Am. J. Trop. Med. Hyg..

[11] Hosoki K., Nakamura A., Kainuma K., Sugimoto M., Nagao M., Hiraguchi Y., Tanida H., Tokuda R., Wada H., Nobori T. (2013). Differential Activation of Eosinophils by Bacteria Associated with Asthma. Int. Arch. Allergy Immunol..

[12] Covian F.L. (1977). Eosinophils. III. Phagocytosis. Rev. Clin. Espanola.

[13] Cohen S.G., Sapp T.M. (1969). Phagocytosis of bacteria by eosinophils in infectious-related asthma. J. Allergy.

[14] Gestal M.C., Blas-Machado U., Johnson H.M., Rubin L.N., Dewan K.K., Bryant C., Tiemeyer M., Harvill E.T. (2020). Disrupting Bordetella Immunosuppression Reveals a Role for Eosinophils in Coordinating the Adaptive Immune Response in the Respiratory Tract. Microorganisms.

[15] Yazdanbakhsh M., Eckmann C.M., Bot A.A., Roos D. (1986). Bactericidal action of eosinophils from normal human blood. Infect. Immun..

[16] Cline M.J. (1972). Microbicidal activity of human eosinophils. J. Reticuloendothel. Soc..

[17] Mickenberg I.D., Root R.K., Wolff S.M. (1972). Bactericidal and metabolic properties of human eosinophils. Blood.

[18] George S.M., Karanovic S., Harrison D.A., Rani A., Birnie A.J., Bath-Hextall F.J., Ravenscroft J.C., Williams H.C. (2019). Interventions to reduce Staphylococcus aureus in the management of eczema. Cochrane Database Syst. Rev..

[19] Hosoki K., Nakamura A., Nagao M., Hiraguchi Y., Tanida H., Tokuda R., Wada H., Nobori T., Suga S., Fujisawa T. (2012). Staphylococcus aureus directly activates eosinophils via platelet-activating factor receptor. J. Leukoc. Biol..

[20] Prince L.R., Graham K.J., Connolly J., Anwar S., Ridley R., Sabroe I., Foster S.J., Whyte M.K.B. (2012). Staphylococcus aureus Induces Eosinophil Cell Death Mediated by α-hemolysin. PLoS One.

[21] Gevaert E., Zhang N., Krysko O., Lan F., Holtappels G., De Ruyck N., Nauwynck H., Yousefi S., Simon H.-U., Bachert C. (2017). Extracellular eosinophilic traps in association with Staphylococcus aureus at the site of epithelial barrier defects in patients with severe airway inflammation. J. Allergy Clin. Immunol..

[22] Cowardin C.A., Buonomo E.L., Saleh M.M., Wilson M.G., Burgess S.L., Kuehne S.A., Schwan C., Eichhoff A.M., Koch-Nolte F., Lyras D. (2016). The binary toxin CDT enhances Clostridium difficile virulence by suppressing protective colonic eosinophilia. Nat. Microbiol..

[23] Arnold I.C., Artola-Borán M., Tallón de Lara P., Kyburz A., Taube C., Ottemann K., van den Broek M., Yousefi S., Simon H.-U., Müller A. (2018). Eosinophils suppress Th1 responses and restrict bacterially induced gastrointestinal inflammation. J. Exp. Med..

[24] Mai E., Limkar A.R., Percopo C.M., Rosenberg H.F. (2021). Generation of Mouse Eosinophils in Tissue Culture from Unselected Bone Marrow Progenitors. Methods Mol. Biol..

[25] Lee J.J., Jacobsen E.A., Ochkur S.I., McGarry M.P., Condjella R.M., Doyle A.D., Luo H., Zellner K.R., Protheroe C.A., Willetts L. (2012). Human versus mouse eosinophils: “That which we call an eosinophil, by any other name would stain as red.”. J. Allergy Clin. Immunol..

[26] Park Y.M., Bochner B.S. (2010). Eosinophil Survival and Apoptosis in Health and Disease. Allergy Asthma Immunol. Res..

[27] Rothenberg M.E., Mishra A., Brandt E.B., Hogan S.P. (2001). Gastrointestinal eosinophils. Immunol. Rev..

[28] Mishra A., Hogan S.P., Brandt E.B., Rothenberg M.E. (2000). Peyer’s patch eosinophils: identification, characterization, and regulation by mucosal allergen exposure, interleukin-5, and eotaxin. Blood.

[29] Nelson R.K., Bush A., Stokes J., Nair P., Akuthota P. (2020). Eosinophilic Asthma. J. Allergy Clin. Immunol. Pract..

[30] Buhl R., Bel E., Bourdin A., Dávila I., Douglass J.A., FitzGerald J.M., Jackson D.J., Lugogo N.L., Matucci A., Pavord I.D. (2022). Effective Management of Severe Asthma with Biologic Medications in Adult Patients: A Literature Review and International Expert Opinion. J. Allergy Clin. Immunol. Pract..

[31] Melén E., Lambrecht B.N., Lloyd C.M., Rothenberg M.E., Kabashima K., Luciani F., Coquet J.M., Ober C., Nawijn M.C., Platts-Mills T., von Mutius E. (2023). A conversation on allergy: recognizing the past and looking to the future. Immunol. Cell Biol..

[32] Wardlaw A.J., Brightling C., Green R., Woltmann G., Pavord I. (2000). Eosinophils in asthma and other allergic diseases. Br. Med. Bull..

[33] Wechsler M.E., Munitz A., Ackerman S.J., Drake M.G., Jackson D.J., Wardlaw A.J., Dougan S.K., Berdnikovs S., Schleich F., Matucci A. (2021). Eosinophils in Health and Disease: A State-of-the-Art Review. Mayo Clin. Proc..

[34] Zhang S., Caldwell J.M., Rochman M., Collins M.H., Rothenberg M.E. (2024). Machine learning–based identification and characterization of mast cells in eosinophilic esophagitis. J. Allergy Clin. Immunol..

[35] Lilly L.M., Scopel M., Nelson M.P., Burg A.R., Dunaway C.W., Steele C. (2014). Eosinophil Deficiency Compromises Lung Defense against Aspergillus fumigatus. Infect. Immun..

[36] Padigel U.M., Hess J.A., Lee J.J., Lok J.B., Nolan T.J., Schad G.A., Abraham D. (2007). Eosinophils Act as Antigen-Presenting Cells to Induce Immunity to Strongyloides stercoralis in Mice. J. Infect. Dis..

[37] Galioto A.M., Hess J.A., Nolan T.J., Schad G.A., Lee J.J., Abraham D. (2006). Role of Eosinophils and Neutrophils in Innate and Adaptive Protective Immunity to Larval Strongyloides stercoralis in Mice. Infect. Immun..

[38] Gurtner A., Crepaz D., Arnold I.C. (2023). Emerging functions of tissue-resident eosinophils. J. Exp. Med..

[39] Mayer-Barber K.D. (2023). Granulocytes subsets and their divergent functions in host resistance to Mycobacterium tuberculosis — a ‘tipping-point’ model of disease exacerbation. Curr. Opin. Immunol..

[40] Berek C. (2016). Eosinophils: important players in humoral immunity. Clin. Exp. Immunol..

[41] Chu V.T., Beller A., Rausch S., Strandmark J., Zänker M., Arbach O., Kruglov A., Berek C. (2014). Eosinophils Promote Generation and Maintenance of Immunoglobulin-A-Expressing Plasma Cells and Contribute to Gut Immune Homeostasis. Immunity.

[42] Puzzovio P.G., Levi-Schaffer F. (2021). Latest Progresses in Allergic Diseases Biomarkers: Asthma and Atopic Dermatitis. Front. Pharmacol..

[43] Sánchez J., Sánchez A., Munera M., Garcia E., Lopez J.-F., Velásquez-Lopera M., Cardona R. (2021). Presence of IgE Autoantibodies Against Eosinophil Peroxidase and Eosinophil Cationic Protein in Severe Chronic Spontaneous Urticaria and Atopic Dermatitis. Allergy Asthma Immunol. Res..

[44] Kanda A., Yasutaka Y., Van Bui D., Suzuki K., Sawada S., Kobayashi Y., Asako M., Iwai H. (2020). Multiple Biological Aspects of Eosinophils in Host Defense, Eosinophil-Associated Diseases, Immunoregulation, and Homeostasis: Is Their Role Beneficial, Detrimental, Regulator, or Bystander?. Biol. Pharm. Bull..

[45] Rubin K., Glazer S. (2018). The pertussis hypothesis: Bordetella pertussis colonization in the etiology of asthma and diseases of allergic sensitization. Med. Hypotheses.

[46] Diavatopoulos D.A., Cummings C.A., Schouls L.M., Brinig M.M., Relman D.A., Mooi F.R. (2005). Bordetella pertussis, the Causative Agent of Whooping Cough, Evolved from a Distinct, Human-Associated Lineage of B. bronchiseptica. PLoS Pathog..

[47] Arico B., Gross R., Smida J., Rappuoli R. (1987). Evolutionary relationships in the genus Bordetella. Mol. Microbiol..

[48] Gestal M.C., Whitesides L.T., Harvill E.T. (2019). Integrated Signaling Pathways Mediate Bordetella Immunomodulation, Persistence, and Transmission. Trends Microbiol..

[49] Cotter P.A., Miller J.F. (1994). BvgAS-mediated signal transduction: analysis of phase-locked regulatory mutants of Bordetella bronchiseptica in a rabbit model. Infect. Immun..

[50] von Koenig C.H., Tacken A., Finger H. (1988). Use of supplemented Stainer-Scholte broth for the isolation of Bordetella pertussis from clinical material. J. Clin. Microbiol..

[51] McKay L.S., Spandrio A.R., Johnson R.M., Sobran M.A., Marlatt S.A., Mote K.B., Dedloff M.R., Nash Z.M., Julio S.M., Cotter P.A. (2024). Cytochrome oxidase requirements in Bordetella reveal insights into evolution towards life in the mammalian respiratory tract. PLoS Pathog..

[52] Reynolds J. (2005).

[53] First N.J., Pedreira-Lopez J., San-Silvestre M.R.F., Parrish K.M., Lu X.-H., Gestal M.C. (2023). Bordetella spp. utilize the type 3 secretion system to manipulate the VIP/VPAC2 signaling and promote colonization and persistence of the three classical Bordetella in the lower respiratory tract. Front. Cell. Infect. Microbiol..

[54] Wacht G, Poirot A, Charles AL, et al. FACS-based isolation of human eosinophils allows purification of high quuality RNA. J Immunol Methods. 12 2018;463:47-53. doi: 10.1016/j.jim.2018.09.003 10.1016/j.jim.2018.09.003 30217720

